# Brown Carbon in East Asia: Seasonality, Sources, and Influences on Regional Climate and Air Quality

**DOI:** 10.1021/acsenvironau.4c00080

**Published:** 2024-11-13

**Authors:** Fan Wang, Zifeng Lu, Guangxing Lin, Gregory R. Carmichael, Meng Gao

**Affiliations:** † Department of Geography, Hong Kong Baptist University, Hong Kong SAR 999077, China; ‡ Energy Systems and Infrastructure Analysis Division, Argonne National Laboratory, Lemont, Illinois 60439, United States; § College of Ocean and Earth Sciences, Xiamen University, Xiamen 361005, China; ∥ Department of Chemical and Biochemical Engineering, The University of Iowa, Iowa City, Iowa 52242, United States

**Keywords:** brown carbon, WRF-Chem, light-absorbing aerosol, radiative feedback, meteorology, PM_2.5_, O_3_

## Abstract

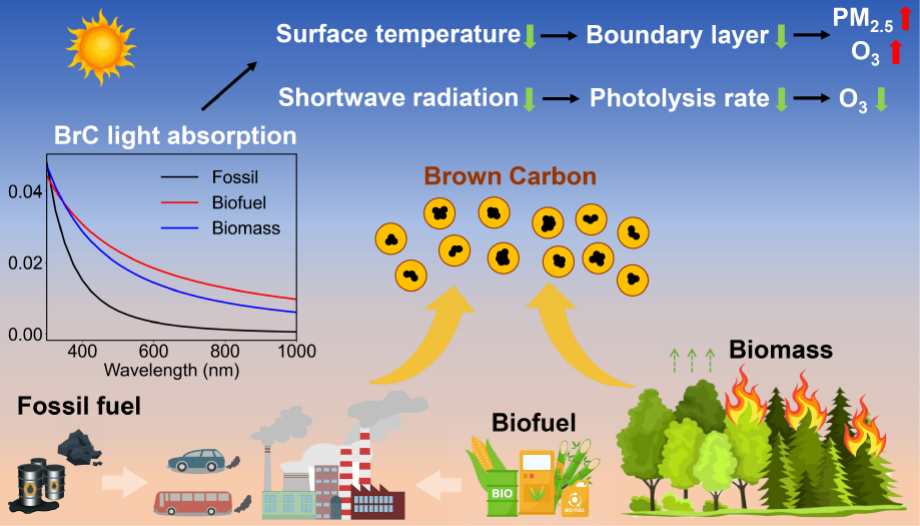

Brown carbon (BrC) has been recognized as an important light-absorbing carbonaceous aerosol, yet understanding of its influence on regional climate and air quality has been lacking, mainly due to the ignorance of regional coupled meteorology-chemistry models. Besides, assumptions about its emissions in previous explorations might cause large uncertainties in estimates. Here, we implemented a BrC module into the WRF-Chem model that considers source-dependent absorption and avoids uncertainties caused by assumptions about emission intensities. To our best knowledge, we made the first effort to consider BrC in a regional coupled model. We then applied the developed model to explore the impacts of BrC absorption on radiative forcing, regional climate, and air quality in East Asia. We found notable increases in aerosol absorption optical depth (AAOD) in areas with high OC concentrations. The most intense forcing of BrC absorption occurs in autumn over Southeast Asia, and values could reach around 4 W m^–2^. The intensified atmospheric absorption modified surface energy balance, resulting in subsequent declines in surface temperature, heat flux, boundary layer height, and turbulence exchanging rates. These changes in meteorological variables additionally modified near-surface dispersion and photochemical conditions, leading to changes of PM_2.5_ and O_3_ concentrations. These findings indicate that BrC could exert important influence in specific regions and time periods. A more in-depth understanding could be achieved later with the developed model.

## 1 Introduction

Carbonaceous aerosols, composed of both black carbon (BC) and organic carbon (OC), have long been believed to affect climate. BC is widely acknowledged as the second most potent warming component after CO_2_;^1^ while OC aerosols were traditionally viewed as cooling components, predominately scattering radiation.^2^ However, a number of subsequent studies identified that certain types of OC aerosols, named brown carbon (BrC), were able to absorb solar radiation, particularly in the near-ultraviolet (300–400 nm) and visible (400–700 nm) ranges.^3^ BrC originates from diverse and intricate sources, including incomplete combustion,^4^ formation of secondary organic aerosols,^5^ and aging processes of aerosols.^6−11^

Ground-based remote sensing measurements revealed the dominant role of BrC in light-absorbing aerosols during late autumn and winter in Beijing.^12^ Yan et al.^13^ also highlighted that residential coal combustion in winter could contribute significantly to BrC in northern China. The ubiquity of BrC in the atmosphere and its strong solar absorption in the ultraviolet and visible ranges suggest its potential significance as a warming aerosol component alongside BC and dust. Previous studies have thoroughly discussed the feedback mechanism of BC on meteorological conditions and pollution during severe haze events in China,^14−16^ yet the influence of BrC on regional climate and air quality has been unexplored. Given the great observed importance of BrC during autumn and winter in China,^12,17^ understanding BrC-radiation interactions is essential for an accurate assessment of the radiative forcing of carbonaceous aerosols in Asia and their influences on regional climate and air quality.

This requires a reasonable description of BrC in climate or chemical transport models. Most models simplify organic aerosols as merely scattering entities, which might lead to discrepancies between modeled and observed aerosol absorption and contribute to large uncertainties in radiative forcing estimates.^18^ Poor understanding of BrC emission inventories is a significant hindrance to model development. While some efforts have provided BrC emissions from specific sources, such as fuel combustion,^19,20^ biomass and coal burning,^21,22^ and open burning and biofuels,^23^ a comprehensive BrC emission inventory is still difficult to obtain. Although some global model studies have explored the global distribution and radiative properties of atmospheric BrC,^23−30^ oversimplified treatment of BrC emissions and properties could introduce uncertainties in the results.

Here, we developed the Weather Research and Forecasting model coupled with Chemistry (WRF-Chem) to consider the optical properties of BrC from different sources and applied it to the region of East Asia to evaluate its effects on regional climate and air quality. In our development, assumptions of emissions of BrC were not needed, which could avoid the associated uncertainties. The research findings address the extant gap in the representation of BrC within regional models and refine our understanding of the interactive mechanisms among aerosols, radiation, and air quality in East Asia.

## 2 Method

### 2.1 WRF-Chem Model Configurations

WRF-Chem version 3.9.1 was adopted here to examine the impacts of BrC absorption on radiation, temperature, air pollutants, etc. Following Gao et al.,^31,32^ a single domain was configured over East Asia with 142 × 105 horizontal grids at a 50 × 50 km resolution and 27 vertical layers. Gas-phase chemistry and aerosol chemistry were simulated with the Carbon-Bond Mechanism version Z (CBMZ)^33^ and the 8-bin version of the Model for Simulating Aerosol Interactions and Chemistry (MOSAIC)^34^ schemes. The RRTM longwave radiation^35^ and the Goddard shortwave radiation^36^ schemes were used to simulate radiative transfer. Other main selected physical schemes included the Noah land surface model,^37^ the Grell 3D Ensemble cumulus scheme,^38^ and the Morrison 2-moment microphysics scheme.^39^ We made modifications to the Yonsei University planetary boundary layer scheme^40^ to output the turbulence exchanging rate.^41^ The 6-hly National Centers of Environmental Prediction (NCEP) final analysis (FNL) data set (https://rda.ucar.edu/datasets/ds083-2/) was adopted as the meteorological initial and boundary conditions. Anthropogenic emissions and biomass burning emissions were taken from the MIX v2 Asian emission inventory of the year 2017, offered on 0.1° × 0.1° grids.^42^ Compared to MIX v1, the MIX v2 Asian emission inventory included emissions of open biomass burning and shipping.^42^ Because the MIX v2 emission inventory only contains OC from anthropogenic processes and biomass burning, the OC emission inventory used to describe BrC absorption properties was obtained from Lu et al.^10,43^ It consists of 1° × 1° global gridded emissions for the year 2010 and was classified into three major sectors: fossil fuel, biofuel, and biomass. Biogenic emissions were generated online by the Model of Emissions of Gases and Aerosols from Nature (MEGAN).^44^ The chemical initial and boundary conditions were provided by daily chemical forecasts from the Community Atmosphere Model with Chemistry (CAM-chem).^45^

We conducted two sets of simulations, named CTRL and NA-OC. CTRL was the control experiment, which accounted for both the scattering and absorbing effects of OC. In the NA-OC simulation, the absorption of shortwave radiation by OC was turned off. By comparing the results of these two experiments, we could evaluate the effects of BrC absorption on radiative forcing, meteorological conditions, and air pollutant concentrations.

### 2.2 Development of BrC Absorption Module in WRF-Chem

In the WRF-Chem model, the optical characteristics of particulate matter are denoted by a complex refractive index (*m* = *n* + *ki*), where *n* represents the real part, indicating scattering properties; and *k* is the imaginary part that stands for absorption properties. We incorporated the wavelength- and source-dependent light-absorbing properties of OC (Table 1 and Figure S1) obtained from Lu et al.^10^ to simulate the influence of BrC absorption. Because fossil fuel-based OC emissions contribute only about 10% of the global budget, and light absorption abilities of BrC from fossil fuels are relatively smaller,^10^ we adopted the total light absorption property of OC from fossil fuels instead of including more detailed sectors. We introduced three tracers into the model to track OC from the combustion of fossil fuels, biofuels, and biomass burning, as well as their vertical mixing, transport, and deposition processes. This approach can reduce uncertainties caused by assumptions about BrC emissions. For example, Lin et al.^27^ assumed that all of the residential coal burning, biomass burning, and the biofuel produced primary organic aerosol (POA) was BrC. Feng et al.^25^ assumed the proportion of BrC in total biofuel and biomass POA to be 66%, while Wang et al.^30^ supposed that 25% of biomass and 50% of biofuel POA were BrC. In addition, the optical properties of aerosols are influenced by particle size distribution and mixing state. We adopted an aerosol module with eight size bins for the particle size distribution. Regarding the mixing state of aerosols, we assumed internal mixing of aerosols, with BC particles acting as the core in a core–shell model and other components as the shell.

**Table 1:** Imaginary Refractive Indices of OC for Wavelengths at 300, 400, 600, and 1000 nm

wavelength	300 nm	400 nm	600 nm	1000 nm
OC from fossil	0.0480	0.015	0.0033	0.0006
OC from biofuel	0.0443	0.0307	0.01843	0.0097
OC from biomass	0.04718	0.02871	0.01436	0.0061

### 2.3 Observations

Daily-averaged air temperature (T_2m_) and relative humidity (RH_2m_) at 2 m, along with wind speed at 10 m (WS_10m_), were taken from weather stations maintained by the China Meteorological Administration (CMA). Surface observations of daily-averaged PM_2.5_ and O_3_ in 2017 were obtained from Air Quality Monitoring Stations operated by the Ministry of Ecology and Environment of the People’s Republic of China. The selection criteria for these data sets required sites to have at least 75% valid data, and the locations of the qualified sites are shown in Figure S2. To assess the accuracy of the simulated aerosol optical depth (AOD) and absorption AOD (AAOD), monthly spectral deconvolution algorithm (SDA) retrievals of AOD and AAOD from the Aerosol Robotic Network (AERONET) in 2017 at level 2 were used. This data set is rigorously quality-controlled, benefiting from both automated cloud screening and manual inspection, as well as pre- and post-field calibration. We applied a second-order polynomial fit method, following Eck et al.,^46^ to interpolate the original AOD and AAOD from AERONET to wavelengths of 300, 400, 600, and 1000 nm, aligning with model's AOD and AAOD outputs.

### 2.4 Uncertainty Analysis

Because AAOD changes were mainly determined by absorbing capacity and OC loading, we developed a random forest network to link imaginary refractive indices and OC emissions with AAOD changes. Prediction models for each wavelength (300, 400, 600, and 1000 nm) were built using the corresponding imaginary refractive indices and emissions of OC from different sources. Each model’s accuracy was evaluated first, and the results showed excellent performance, with R-squared values up to 0.99 (Figure S3). We then used the Monte Carlo approach to perform 10,000 simulations of each model by inputting random imaginary refractive indices and OC emissions within the uncertainty ranges (Table S1). The uncertainty was defined as one standard deviation of all 10,000 outputs and expressed as a percentage.

## 3 Results and Discussion

### 3.1 Model Evaluation

**Figure 1:**
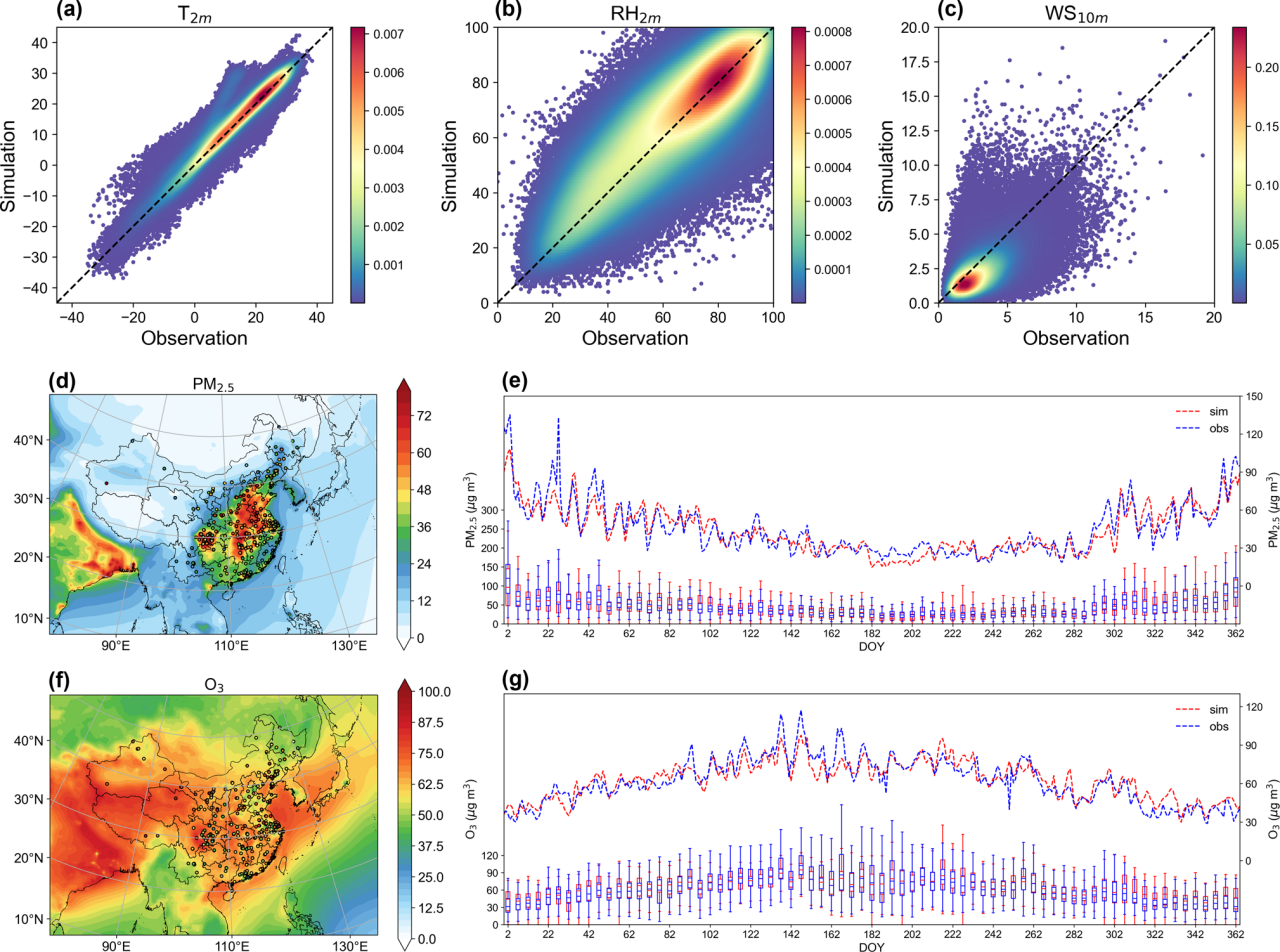
Scatter plots of modeled and observed daily mean surface air temperature (a) and relative humidity (b) at 2 m and wind speed (c) at 10 m. Locations of all the sites can be found in Figure S2a. Spatial distribution of simulated (contour) and observed (dot) annual average surface PM_2.5_ (d) and the concentration of O_3_ (f) concentration. Modeled and observed time series of PM_2.5_ (e) and O_3_ (g) concentrations. Dashed lines indicate average concentrations over all measurement sites. Box plots show distributions of observations and simulations at all the sites.

We evaluated the model performance of the CTRL experiment using observed meteorological variables, air pollutant concentrations, AOD, and AAOD. Daily average T_2m_, RH_2m_, and WS_10m_ improve slightly after considering BrC absorption (Table S2) and are well-captured by the model (Figure 1a–c). The corresponding correlation coefficients with observations reach 0.96, 0.76, and 0.62, respectively. The simulation of WS_10m_ exhibits relatively weaker performance, which is a common issue of the WRF-Chem model that may stem from imprecise land-use data^18,31^ or the used coarse resolution that fails to capture small-scale features influenced by complex topography.^47,48^Figure 1d–g illustrates the observed versus simulated annual spatial and temporal variations of surface PM_2.5_ and O_3_ for the year 2017. The model accurately reproduces the spatial distribution of annual average surface PM_2.5_ and O_3_ across China (Figure 1d,f). Compared to the NA-OC experiment, both PM_2.5_ and O_3_ simulations show decreased bias in the CTRL experiment (Table S2). The seasonality of both pollutants is well-captured, with higher PM_2.5_ concentrations in colder seasons and elevated levels of O_3_ during warmer months of 2017 (Figure 1e,g).

**Figure 2:**
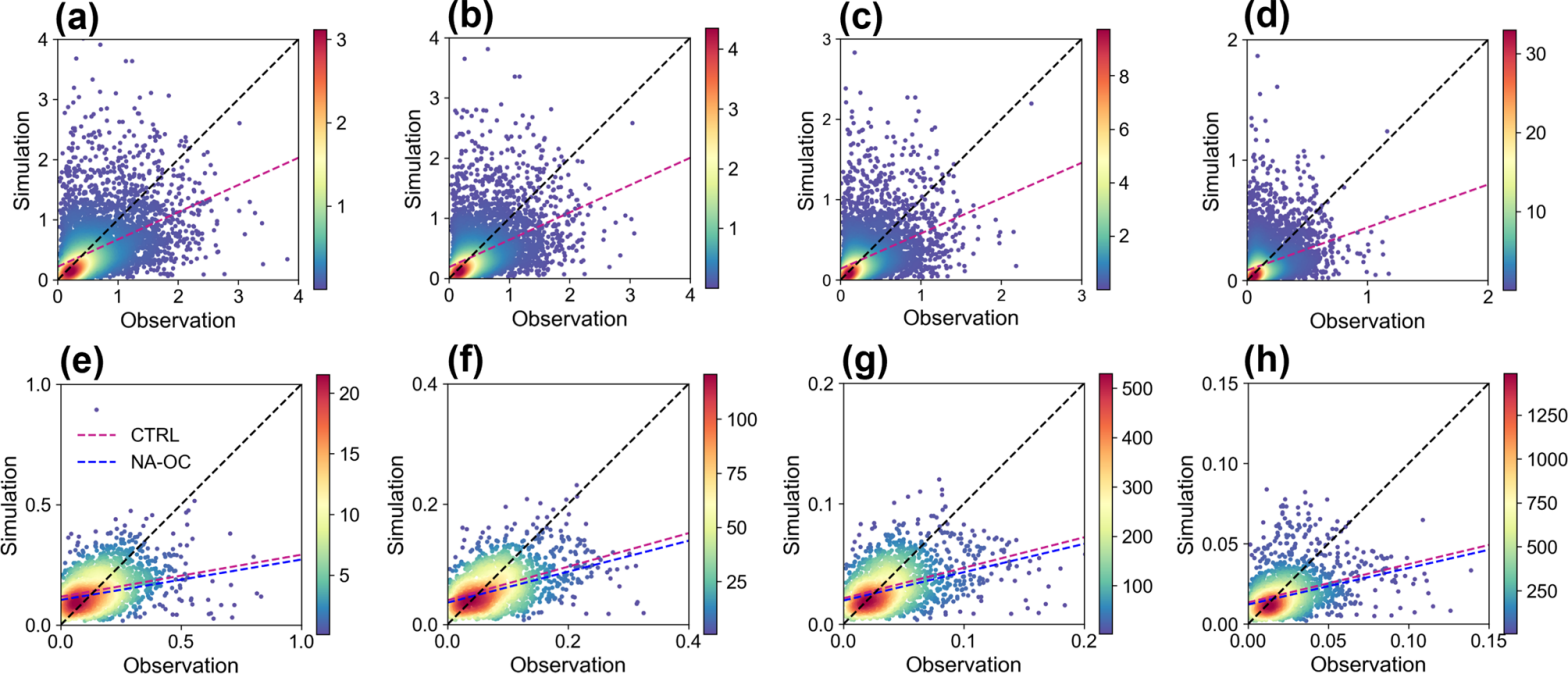
Scatter plots of modeled and observed daily AOD for wavelengths at 300 (a), 400 (b), 600 (c), and 1000 nm (d). Scatter plots of modeled and observed daily AAOD for wavelengths at 300 (e), 400 (f), 600 (g), and 1000 nm (h). Locations of all the sites can be found in Figure S2b.

We also assessed the accuracy of the model in simulating aerosol optical properties by comparing simulated AOD and AAOD at different wavelengths with AERONET measurements. Given the limited temporal and spatial extent of daily AOD and AAOD data from AERONET in our simulation period and domain, we align the model output with the existing observations at each AERONET site in scatter plots (Figure 2).

The comparison reveals good agreement between simulated and observed AOD and AAOD values, with the bulk of the data points situated around the ideal 1:1 line. Disparities found between the model predictions and actual observations could be linked to uncertainties in the emission inventory, the aerosol size distribution assumed for the emissions,^18,49^ etc. Although improvement in AOD simulation is not evident, incorporating BrC absorption partially offsets the underestimation of AAOD (Figure 2e–h). The average AAOD increased by 0.0144 (12.6%), 0.00564 (11.5%), 0.00252 (9.92%), and 0.00158 (9.63%) at wavelengths of 300, 400, 600, and 1000 nm, respectively, after considering BrC absorption in the model.

### 3.2 Impacts of BrC Absorption on Shortwave Radiative Forcing

**Figure 3:**
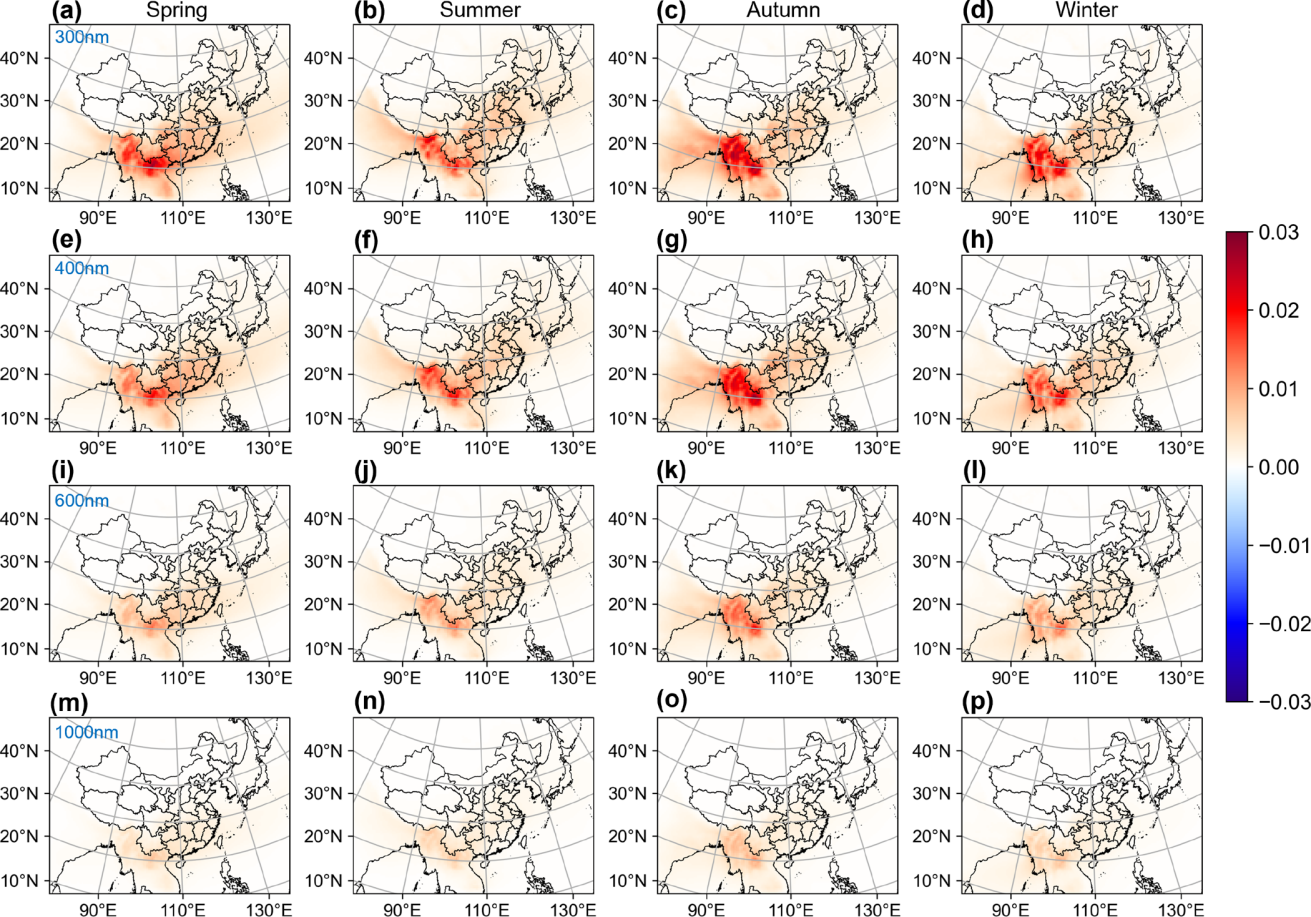
Spatial distribution of difference in seasonal mean AAOD for wavelengths at 300 (a–d), 400 (e–h), 600 (i–l), and 1000 nm (m–p) between CTRL and NA-OC (CTRL minus NA-OC).

Figure S4 shows the spatial distribution of modeled OC from three major sources. Fossil fuel and biofuel combustion are two primary contributors to total OC loading in China. These two sources are highly associated with human activities and thus the distribution of population. The majority of biomass source OC is located in Southeast Asia. In accordance with the spatial distribution of OC, the inclusion of BrC absorption in the model markedly alters the modeled annual average AAOD over these regions with high OC burden (Figure S5a). BrC absorption increases the AAOD at 300 nm by approximately 0.01 over East and South China. More pronounced changes are evident in Southeast Asia, with an annual average increase between 0.02 and 0.03 (Figure S5a), which can be attributed to the high biomass burning emission rates and the fact that OC from biomass burning has a greater light absorption capability compared to other sources (Table 1). Figure 3 shows differences in AAOD at wavelengths of 300, 400, 600, and 1000 nm between the CTRL and NA-OC experiments across all four seasons. The most significant AAOD changes occur at 300 and 400 nm, where BrC exhibits higher absorption efficiency (Table 1). The greatest AAOD increase reaches about 0.03 in Southeast Asia during autumn and winter (Figure 3). The average uncertainties of AAOD changes are 38.86, 40.47, 45.06, and 52.80% at wavelengths of 300, 400, 600, and 1000 nm, respectively.

**Figure 4:**
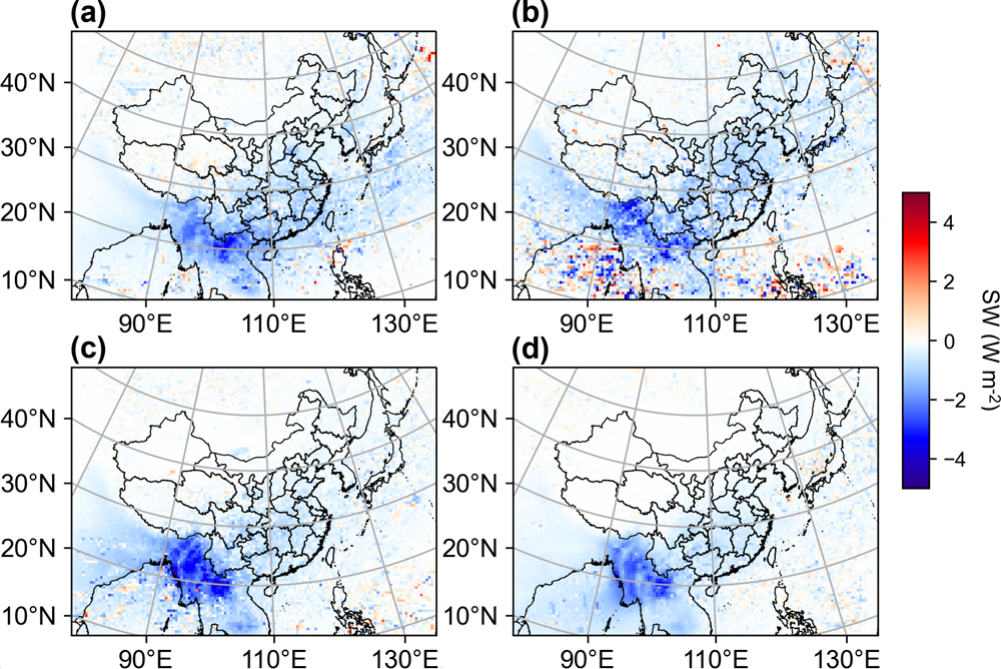
Spatial distribution of difference in seasonal mean surface SW in spring (a), summer (b), autumn (c), and winter (d) between CTRL and NA-OC (CTRL minus NA-OC).

Due to the absorption of OC, downward shortwave radiation (SW) is also lower in the CTRL experiment (Figure S5b). The BrC absorption-induced annual average shortwave radiative forcing is approximately 0.5–2 W m^–2^ over East, Central, and East China, while the most substantial regional radiative forcing, reaching 2–4 W m^–2^ annually, occurs over Southeast Asia. The radiative effect of BrC absorption peaks in spring and summer over East and South China, approaching 1 W m^–2^ (Figure 4a,b). The most intense forcing of BrC absorption occurs in autumn over Southeast Asia, and values could reach around 4 W m^–2^ (Figure 4c).

Radiative forcing of BrC has also been explored previously by using several global models. For instance, Saleh et al.^29^ estimated the annual average direct radiative effect (DRE) of OC absorption in 2005 to be approximately 1 W m^–2^ over Southeast Asia and 0.5–1 W m^–2^ in South and East China using the GEOS-Chem model. Zhang et al.^50^ calculated an annual average DRE of BrC absorption in 2010 at 1 W m^–2^ over Southeast Asia, and roughly 0.3 W m^–2^ in China with the Community Earth System Model (CESM). Brown et al.^24^ simulated the radiative forcing due to BrC absorption to be 1.5 W m^–2^ over Southeast Asia and 0.5–1 W m^–2^ in South and East China from 2003 to 2011 using the Community Atmosphere Model (CAM5). These estimates derived from global models are comparable with our findings in China because total OC loadings are relatively small in China (Figure 3a). Nonetheless, there are notable differences over Southeast Asia, where our inferred BrC burden is substantially higher. The primary distinction between our simulation and prior model assessments lies in the light-absorbing characteristics of BrC and the BrC emission inventory, underscoring the need for a more comprehensive understanding of optical parameters and the accuracy of emission inventories in future research.^51^

### 3.3 Impacts on Meteorological Conditions

**Figure 5:**
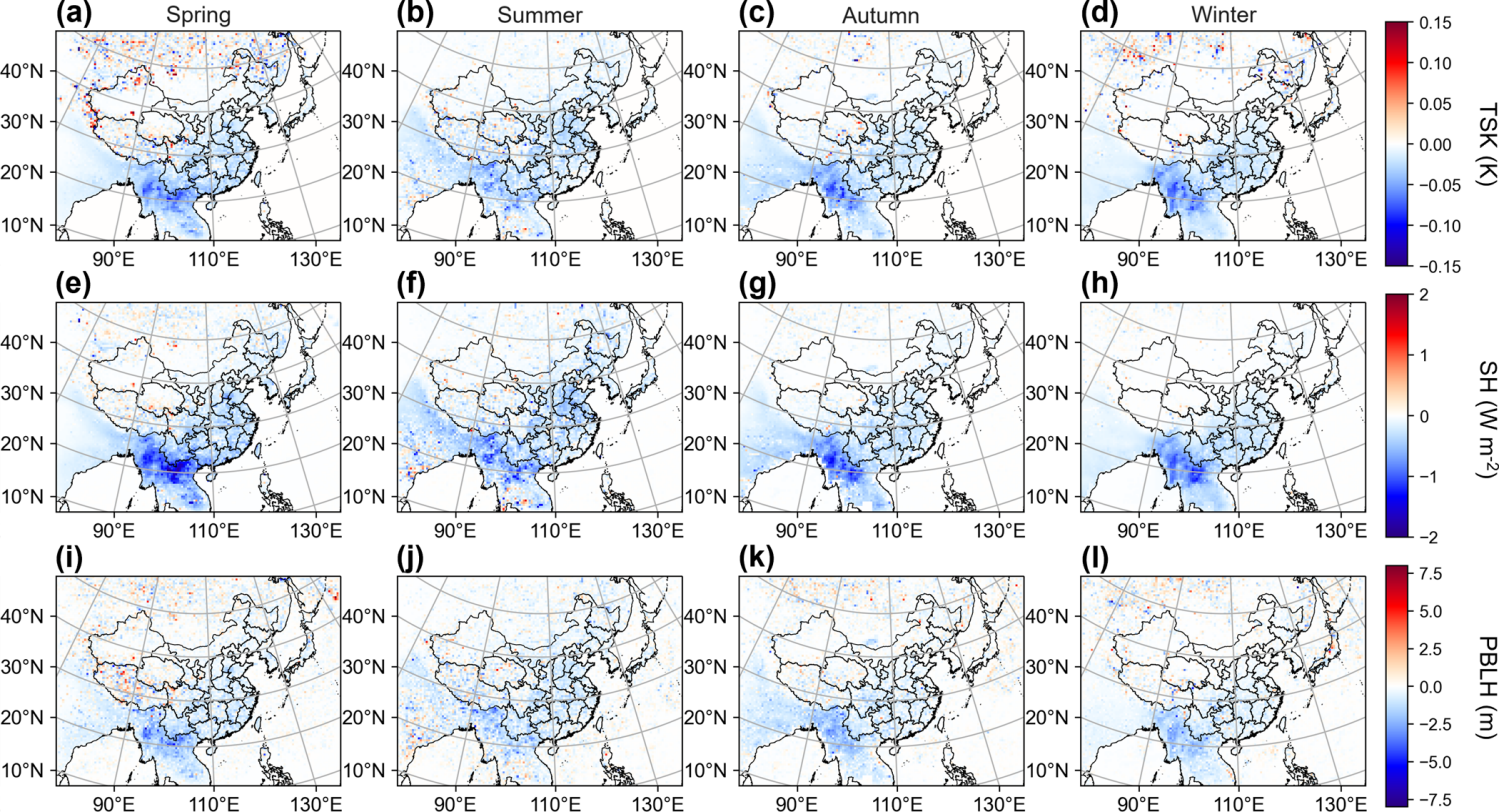
Spatial distribution of difference in seasonal mean TSK (a–d), SH (e–h), and PBLH (i–l) between CTRL and NA-OC (CTRL minus NA-OC).

Radiative forcing of BrC absorption has a substantial influence on the surface radiation balance, which, in turn, affects surface meteorological conditions. Figure 5 shows the responses of surface temperature (TSK), sensible heat (SH), planet boundary layer height (PBLH), and turbulence exchanging rates (EXCH) to BrC absorption. These variables exhibit spatial congruence with the radiative effects of BrC absorption, with marked effects observed in East and South China, as well as Southeast Asia. The impact on TSK is particularly evident during spring and autumn, with the most significant decreases exceeding 0.1 K (Figure 5a,c). The influence is less pronounced during summer, when stronger solar radiation and better OC dispersion conditions prevail (Figure 5b). Surface energy flux also exhibits different seasonal patterns modulated by BrC absorption (Figure 5e–h). Notably, SH changes mostly in spring, with the highest increases surpassing 2 W m^–2^ over Southeast Asia, significantly differing from the patterns observed in the other three seasons. This seasonal variation correlates with shifts in rainfall patterns in this region. The spring dry season in Southeast Asia leads to reduced soil moisture,^52^ rendering SH more susceptible to changes in radiation.^53^ As surface SH flux is the primary energy contributor to boundary layer development, the PBLH experiences its most pronounced reduction, around 5 min, during spring (Figure 5i).

The influence of BrC absorption on surface meteorological conditions, although notable, is significantly less substantial when compared to the absorption feedback from BC. Our previous investigation into the impacts of BC absorption on surface air temperature demonstrated a temperature decrease ranging from 0.5 to 1 °C in the North China Plain.^54^ Global simulations of the radiative effects of BC and BrC suggest that BC accounts for 41% of the total DRE of aerosols, while the contribution of BrC is relatively minor, ranging between 4 and 11%.^25^ Nevertheless, the role of BrC should not be underestimated, as every additional 0.1 °C of warming can cause clearly discernible increases in the intensity and frequency of temperature and precipitation extremes, as well as agricultural and ecological droughts in certain regions under the background of climate change.^55^ Given the primary contribution of biomass burning to BrC in Southeast Asia, along with the increasing intensity of wildfires observed in recent years,^56,57^ it is reasonable to postulate that the radiative forcing effects of BrC could be largely enhanced in the future.

### 3.4 Impacts on Surface PM_2.5_ and O_3_ Concentration

Changes in meteorological conditions such as temperature, mixing rate, and radiation exert profound effects on surface air quality. We found a general increase in PM_2.5_ levels throughout the whole study domain resulting from light absorption by BrC (Figure S6). This can be attributed to the weakened vertical mixing (Figure S7). Conversely, O_3_ concentrations do not exhibit a uniform increase across the whole domain. O_3_ rises over Southeast Asia and South China but diminishes in East China. This pattern is potentially due to the conflicting consequences of diminished dispersion capacity and inhibited photochemical reactions (Figure S7).

**Figure 6:**
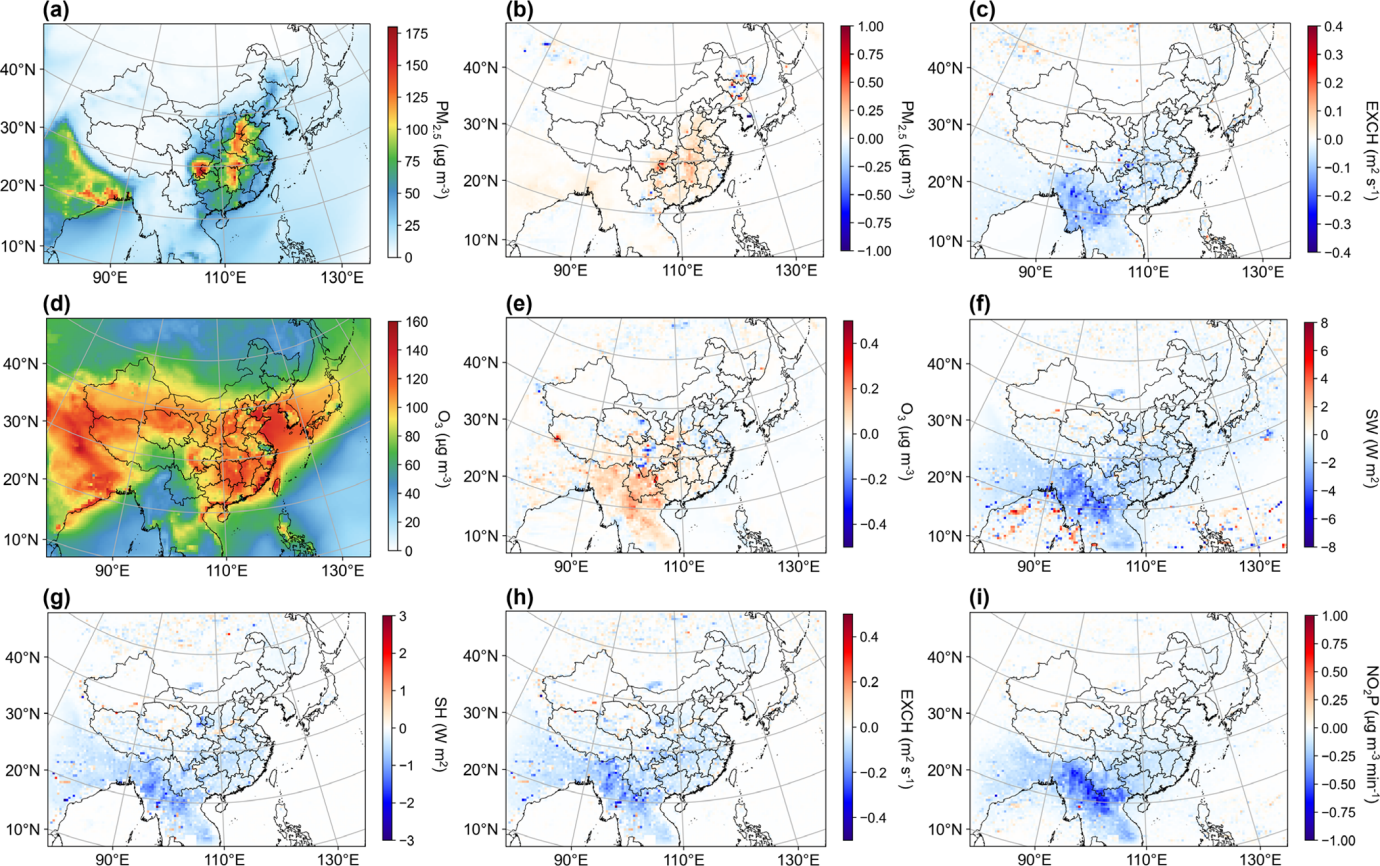
Spatial distribution of monthly mean surface PM_2.5_ (a) in January and O_3_ (d) in September; difference in monthly mean surface PM_2.5_ (b) and EXCH (c) in January; surface O_3_ (e), SW (f), SH (g), EXCH (h), and NO_2_P (i) in September between CTRL and NA-OC (CTRL minus NA-OC).

We further assessed the changes in surface PM_2.5_ in January and O_3_ in September affected by BrC absorption, coinciding with the peak monthly concentrations of these pollutants and their related physicochemical conditions (Figure 6). In January, high PM_2.5_ concentrations are prevalent over Central China and the Sichuan Basin (Figure 6a). The BrC absorption effect cuts down surface SW, which, in turn, reduces the EXCH of 0.1–0.4 m^–2^ s^–1^ in these regions (Figure 6c). As a result, PM_2.5_ increases by 0.25–0.5 μg m^–3^ in central, southern, and eastern parts of China with the most pronounced enhancement of nearly 1 μg m^–3^ in the Sichuan Basin (Figure 6b). The impact of BrC absorption on the oxophore on O_3_ is more complex. We observe an increase of around 0.2 μg m^–3^ in the O_3_ concentration in the southern regions but a slight decrease of ∼0.1 μg m^–3^ over the North China Plain (NCP). While the reduction in downward SW radiation weakens vertical mixing within the boundary layer (Figure 6h), it concurrently decelerates the photolysis rate of NO_2_ (Figure 6i). The dual impacts of unfavorable dispersion conditions and depressed photochemical reactions lead to spatially heterogeneous effects of BrC absorption on surface O_3_ concentration (Figure 6e).

## 4 Summary

While some global models have considered the effects of BrC absorption, the assumptions of BrC emissions could lead to large uncertainties. Besides, the effects of BrC on regional climate and air quality remain unclear. Here, we developed the widely used WRF-Chem model to include the absorption of OC and examine its seasonality, source characteristics, and influences on the regional climate and air quality of East Asia. We find that the absorption of BrC notably enhances the AAOD in regions with high OC concentrations. The increased atmospheric absorption modifies the surface energy balance, resulting in subsequent declines in surface temperature, heat flux, boundary layer height, and turbulence exchanging rates. These changes in meteorological variables additionally modify near-surface dispersion and photochemical conditions, leading to changes of PM_2.5_ and O_3_ concentrations. We also find spatially different influences of BrC on O_3_ concentrations, due to the competing effects of diminished dispersion capacity and inhibited photochemical reactions. These findings suggest that BrC plays a non-negligible role in regional climate and air quality, necessitating additional attention to BrC in regional weather and climate modeling.

We conducted an initial effort to quantify the impact of BrC absorption in the WRF-Chem model, enabling the community to assess the effects of BrC absorption on radiation, regional weather, and air quality. The primary challenge in modeling BrC absorption effects is the uncertainties in the BrC emission inventory and the differentiation of OC from various emission sources.^51,58^ We introduced three new species into the model and incorporated them solely for the calculation of optical properties to mitigate these issues. However, our results also depend on the accuracy of the OC emission inventory, although the uncertainties are generally lower than the previous estimates of BrC emissions.^23−25,27,29,30,50,59^ This can be further improved if observations are available to constrain modeled OC or OC emission intensity.

Given the uncertainties in imaginary refractive indices and emission intensity, as indicated by Lu et al.,^10,43^ the uncertainties in modeled changes in meteorological variables and air pollutant concentrations should be considered. The WRF-Chem model is a complex numerical model, and ensemble simulations have long been considered the most effective method to estimate uncertainties in model outputs,^60−62^ though they are resource- and time-consuming. Considering the strong connection between AAOD changes and BrC absorption indices and loading, we quantify the uncertainty of simulated changes in AAOD after considering BrC absorption using a combination of the random forest network and Monte Carlo methods. We attempted to extend this method to other variables but obtained unreasonable results. Future efforts are required to conduct multiple simulations to determine the uncertainties in other meteorological variables and air pollutants for a more robust evaluation.

Although considering that BrC absorption can reduce model errors in simulating AAOD, our current model still systematically underestimates AAOD, which may be caused by missing secondary formation and aging processes. Secondary formation through oxidation of organic precursors^63−66^ and aging processes due to oxidation,^67,68^ evaporation,^69^ and volatilization^70,71^ can enhance its absorbing properties. Saleh et al.^72^ measured a stronger absorbing compacity of secondary BrC than primary BrC in the short visible and near-ultraviolet ranges. Gao et al.^73^ reported that secondary BrC accounted for 87% of the total BrC absorption at a mountain station in summer. Li et al.^74^ observed a 30% enhancement in BrC's contribution to total absorbance in the semiurban environment of Beijing. Calderon-Arrieta et al.^69^ also revealed a 2-fold enhancement of BrC light absorption due to aging through evaporation of volatile components. These results suggest that including the secondary formation and aging processes of BrC, which have been widely ignored, has significant potential to improve the simulation of AAOD in coupled meteorology-chemistry models. Previous efforts have added the secondary formation and aging processes of BrC in global models,^24,75,76^ but due to limited observations, these models have not been well-constrained. We summarize existing observations and laboratory measurements and incorporate appropriate BrC secondary formation and aging processes into regional models to further improve their capability in simulating BrC optical properties.

BrC is a less potent absorbing component of aerosols compared to BC, which results in relatively minor effects of BrC absorption on the Earth’s system.^25,30^ However, the influence can be important in specific regions and time periods. For example, Southeast Asia, South America, and North America exhibit the highest global mean surface BrC concentrations,^58^ and these regions face an escalating wildfire risk due to global warming.^77−79^ With our developed model, a more in-depth understanding of the influence of BrC could be achieved later.

## Supporting Information Available

Figures of the imaginary refractive indices, locations of observation sites, validation of predicted AAOD, spatial distribution of annual average total OC burden, difference in annual averaged AAOD, and difference in seasonal mean surface PM_2.5_ and O_3_ (PDF)
