# Supplementary material for: Brown Carbon in East Asia: Seasonality, Sources, and Influences on Regional Climate and Air Quality
Source: ACS Environ Au. 2024 Nov 13;5(1):128–37. doi: 10.1021/acsenvironau.4c00080 (PMC11741057; doi:10.1021/acsenvironau.4c00080)
Supplement: Supplementary file 1 — vg4c00080_si_001.pdf [file vg4c00080_si_001.pdf]

Supplementary Materials for

Brown carbon in East Asia: seasonality, sources and influences on regional climate and air quality

Fan Wang[1], Zifeng Lu[2], Guangxing Lin[3], Gregory R. Carmichael[4], Meng Gao[1,*]

1 Department of Geography, Hong Kong Baptist University, Hong Kong SAR, 999077, China,

2 Energy Systems and Infrastructure Analysis Division, Argonne National Laboratory, Lemont, Illinois, 60439, United States of America

3 College of Ocean and Earth Sciences, Xiamen University, Xiamen, 361005, China

4 Department of Chemical and Biochemical Engineering, The University of Iowa, Iowa City, Iowa, 52242, USA

* Corresponding Author: Meng Gao (mmgao2@hkbu.edu.hk).

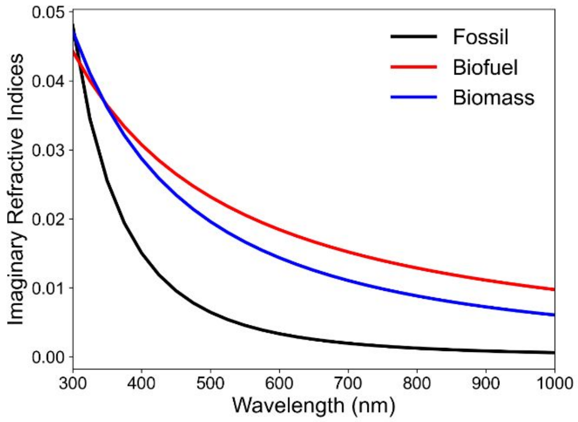

Fig. S1. The imaginary refractive indices of OC for the wavelength.

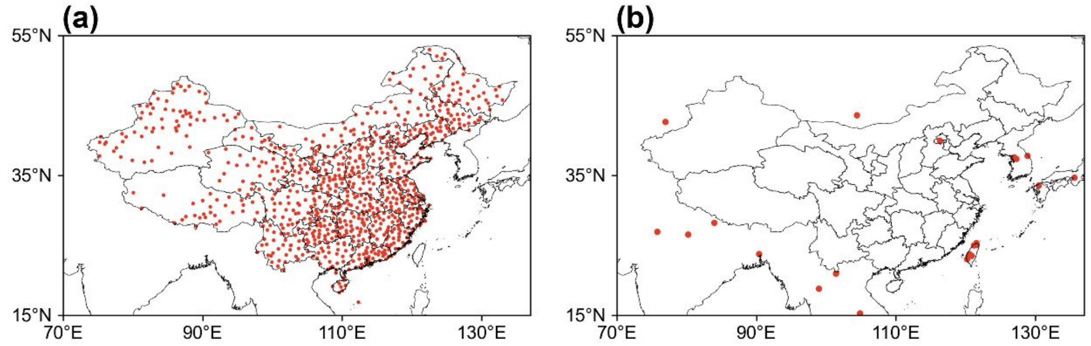

Fig. S2. Locations of weather stations (a) and AOD observation sites from AERONET (b).

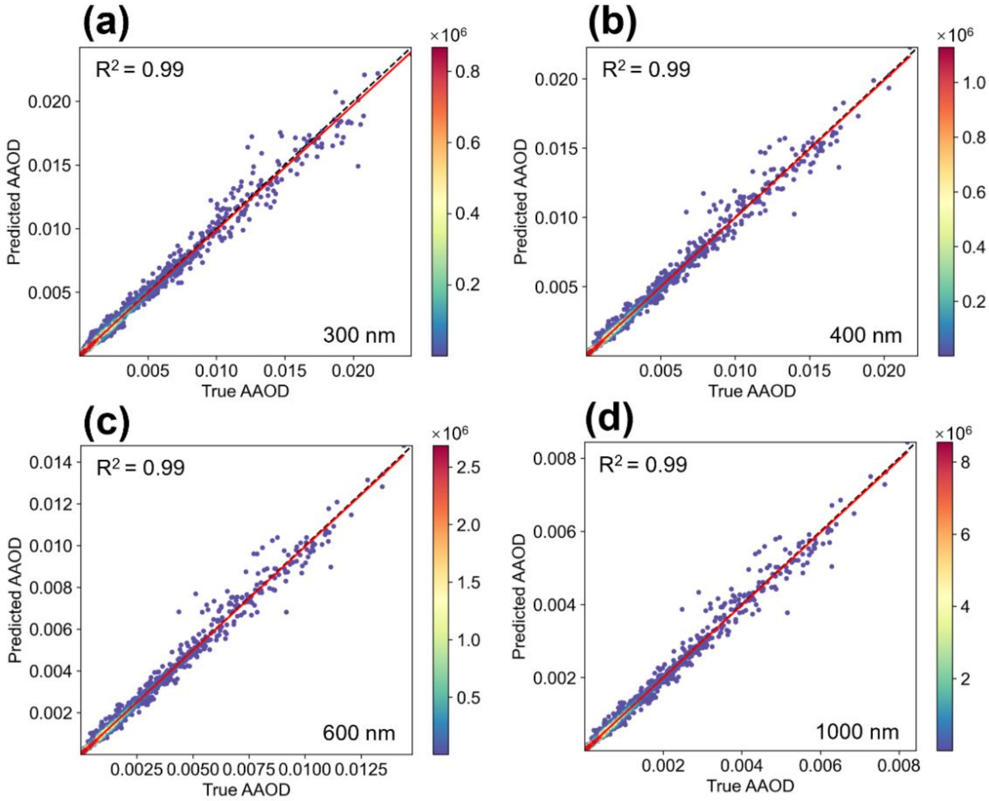

Fig. S3. Validation of predicted AAOD at wavelengths of 300 nm (a), 400 nm (b), 600 nm (c) and 1000 nm (d).

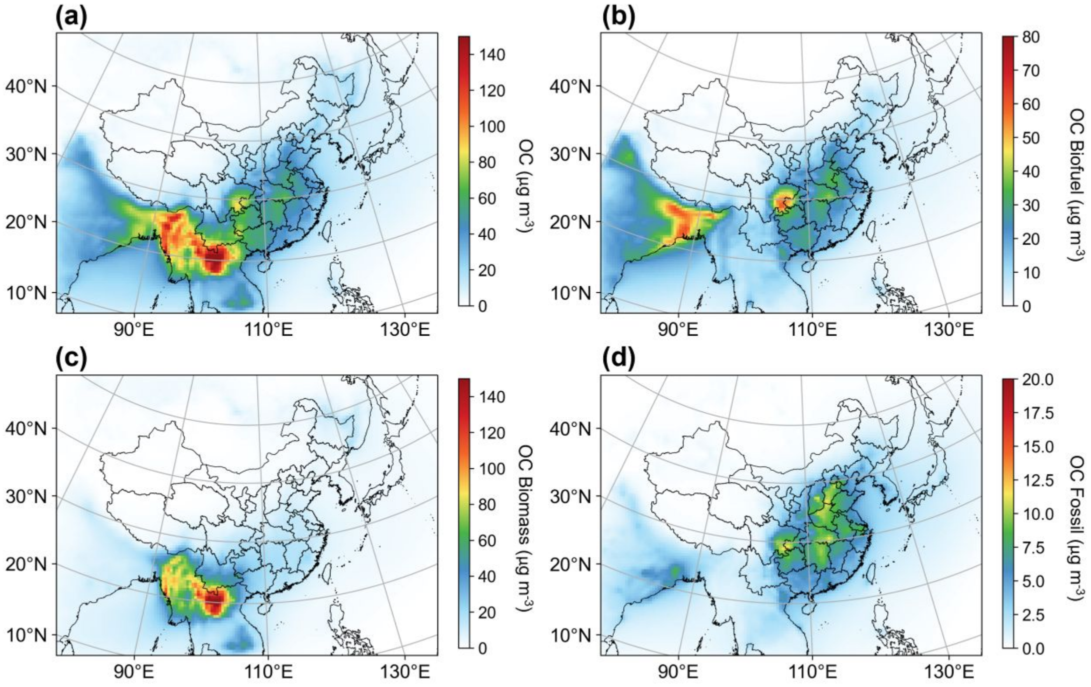

Fig. S4. Spatial distribution of annual average total OC burden (a), OC burden from biofuel (b), biomass (c) and fossil combustion (d) in the atmosphere adopted to calculate BrC absorption properties.

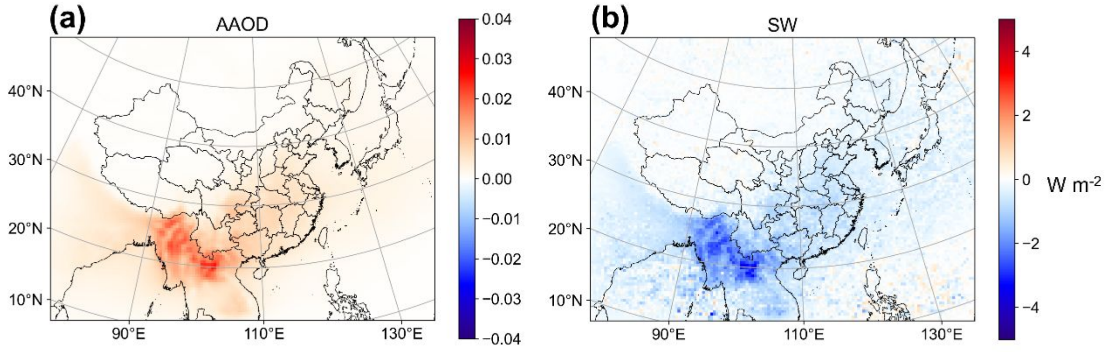

Fig. S5. Spatial distribution of difference in annual averaged AAOD at 300 nm (a) and surface SW (b) between CTRL and NA-OC (CTRL minus NA-OC).

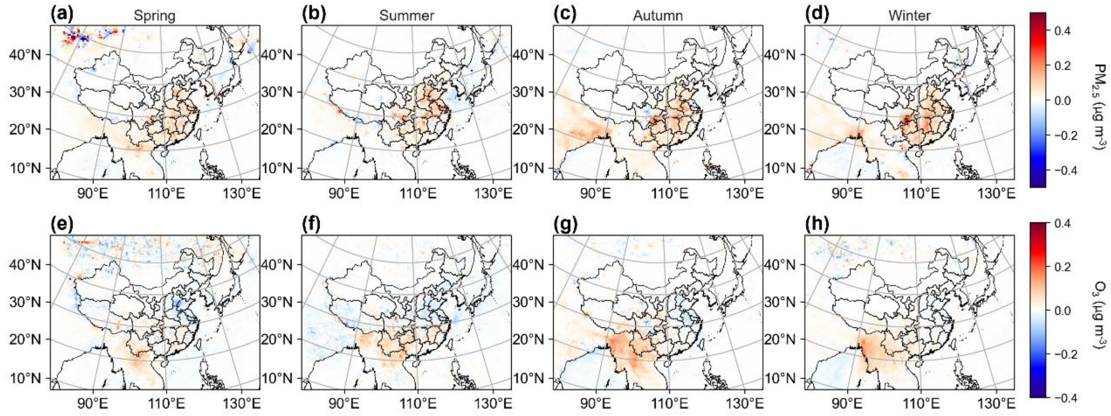

Fig. S6. Spatial distribution of difference in seasonal mean surface $PM_{2.5}$ (a-d) and O_3 (e-h) concentration between CTRL and NA-OC (CTRL minus NA-OC).

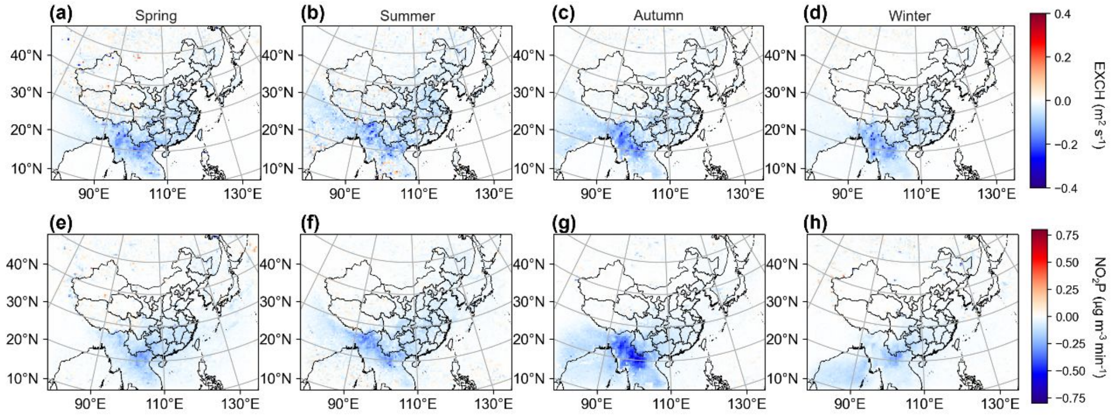

Fig. S7. Spatial distribution of difference in seasonal mean EXCH (a-d) and NO_2P (e-h) between CTRL and NA-OC (CTRL minus NA-OC).

Table S1 Uncertainties of imaginary refractive indices and emissions of OC from different sources

	300 nm	400 nm	600 nm	1000 nm	Emission
Fossil	±66.00%	±65.75%	±65.90%	±68.00%	-68% - 206%
Biofuel	±30.54%	±33.54%	±45.82%	±67.08%	-66% - 143%
Biomass	±32.31%	±36.70%	±49.72%	±71.07%	-49% - 75%

Table S2 Correlation coefficient (R), root mean square error (RMSE), and mean error (ME) between observations and simulations from CTRL and NO-OC experiments.

	T_{2m}			RH_{2m}			WS_{10m}			$PM_{2.5}$			O_3		
	R	RMSE	ME	R	RMSE	ME	R	RMSE	ME	R	RMSE	ME	R	RMSE	ME
CRTL	0.96	3.55	2.32	0.76	21.35	11.69	0.62	1.42	1.07	0.89	9.41	7.01	0.89	8.11	6.24
NA-OC	0.96	3.55	2.33	0.76	21.35	11.70	0.61	1.43	1.07	0.89	9.42	7.02	0.89	8.12	6.24
